# Supplementary material for: Iterative improvement in the automatic modular design of robot swarms
Source: PeerJ Comput Sci. 2020 Dec 7;6:e322. doi: 10.7717/peerj-cs.322 (PMC7924708; doi:10.7717/peerj-cs.322)
Supplement: Supplemental Information 3 [file peerj-cs-06-322-s003.zip › argos3/doc/api/standalone/a00465_source.html]

ARGoS: plugins/robots/foot-bot/simulator/footbot\_entity.h Source File


- Main Page
- Related Pages
- Namespaces
- Classes
- Files

- File List
- File Members

# plugins/robots/foot-bot/simulator/footbot\_entity.h

Go to the documentation of this file.

```
00001 
00007 #ifndef FOOTBOT_ENTITY_H
00008 #define FOOTBOT_ENTITY_H
00009 
00010 namespace argos {
00011    class CControllableEntity;
00012    class CFootBotDistanceScannerEquippedEntity;
00013    class CEmbodiedEntity;
00014    class CFootBotEntity;
00015    class CFootBotTurretEntity;
00016    class CGripperEquippedEntity;
00017    class CGroundSensorEquippedEntity;
00018    class CLEDEquippedEntity;
00019    class CLightSensorEquippedEntity;
00020    class COmnidirectionalCameraEquippedEntity;
00021    class CPerspectiveCameraEquippedEntity;
00022    class CProximitySensorEquippedEntity;
00023    class CRABEquippedEntity;
00024    class CWiFiEquippedEntity;
00025 }
00026 
00027 #include <argos3/core/simulator/entity/composable_entity.h>
00028 #include <argos3/plugins/simulator/entities/wheeled_entity.h>
00029 
00030 namespace argos {
00031 
00032    class CFootBotEntity : public CComposableEntity {
00033 
00034    public:
00035 
00036       ENABLE_VTABLE();
00037 
00038    public:
00039 
00040       CFootBotEntity();
00041 
00042       CFootBotEntity(const std::string& str_id,
00043                      const std::string& str_controller_id,
00044                      const CVector3& c_position = CVector3(),
00045                      const CQuaternion& c_orientation = CQuaternion(),
00046                      Real f_rab_range = 3.0f,
00047                      size_t un_rab_data_size = 10,
00048                      const CRadians& c_omnicam_aperture = ToRadians(CDegrees(70.0f)),
00049                      bool b_perspcam_front = true,
00050                      const CRadians& c_perspcam_aperture = ToRadians(CDegrees(30.0f)),
00051                      Real f_perspcam_focal_length = 0.035,
00052                      Real f_perspcam_range = 2.0);
00053 
00054       virtual void Init(TConfigurationNode& t_tree);
00055       virtual void Reset();
00056       virtual void UpdateComponents();
00057       
00058       inline CControllableEntity& GetControllableEntity() {
00059          return *m_pcControllableEntity;
00060       }
00061 
00062       inline CFootBotDistanceScannerEquippedEntity& GetDistanceScannerEquippedEntity() {
00063          return *m_pcDistanceScannerEquippedEntity;
00064       }
00065 
00066       inline CFootBotTurretEntity& GetTurretEntity() {
00067          return *m_pcTurretEntity;
00068       }
00069 
00070       inline CEmbodiedEntity& GetEmbodiedEntity() {
00071          return *m_pcEmbodiedEntity;
00072       }
00073 
00074       inline CGripperEquippedEntity& GetGripperEquippedEntity() {
00075          return *m_pcGripperEquippedEntity;
00076       }
00077 
00078       inline CGroundSensorEquippedEntity& GetGroundSensorEquippedEntity() {
00079          return *m_pcGroundSensorEquippedEntity;
00080       }
00081 
00082       inline CLEDEquippedEntity& GetLEDEquippedEntity() {
00083          return *m_pcLEDEquippedEntity;
00084       }
00085 
00086       inline CLightSensorEquippedEntity& GetLightSensorEquippedEntity() {
00087          return *m_pcLightSensorEquippedEntity;
00088       }
00089 
00090       inline COmnidirectionalCameraEquippedEntity& GetOmnidirectionalCameraEquippedEntity() {
00091          return *m_pcOmnidirectionalCameraEquippedEntity;
00092       }
00093 
00094       inline CPerspectiveCameraEquippedEntity& GetPerspectiveCameraEquippedEntity() {
00095          return *m_pcPerspectiveCameraEquippedEntity;
00096       }
00097 
00098       inline CProximitySensorEquippedEntity& GetProximitySensorEquippedEntity() {
00099          return *m_pcProximitySensorEquippedEntity;
00100       }
00101 
00102       inline CRABEquippedEntity& GetRABEquippedEntity() {
00103          return *m_pcRABEquippedEntity;
00104       }
00105 
00106       inline CWheeledEntity& GetWheeledEntity() {
00107          return *m_pcWheeledEntity;
00108       }
00109 
00110       inline CWiFiEquippedEntity& GetWiFiEquippedEntity() {
00111          return *m_pcWiFiEquippedEntity;
00112       }
00113 
00114       virtual std::string GetTypeDescription() const {
00115          return "foot-bot";
00116       }
00117 
00118    private:
00119 
00120       CControllableEntity*                   m_pcControllableEntity;
00121       CFootBotDistanceScannerEquippedEntity* m_pcDistanceScannerEquippedEntity;
00122       CFootBotTurretEntity*                  m_pcTurretEntity;
00123       CEmbodiedEntity*                       m_pcEmbodiedEntity;
00124       CGripperEquippedEntity*                m_pcGripperEquippedEntity;
00125       CGroundSensorEquippedEntity*           m_pcGroundSensorEquippedEntity;
00126       CLEDEquippedEntity*                    m_pcLEDEquippedEntity;
00127       CLightSensorEquippedEntity*            m_pcLightSensorEquippedEntity;
00128       COmnidirectionalCameraEquippedEntity*  m_pcOmnidirectionalCameraEquippedEntity;
00129       CPerspectiveCameraEquippedEntity*      m_pcPerspectiveCameraEquippedEntity;
00130       CProximitySensorEquippedEntity*        m_pcProximitySensorEquippedEntity;
00131       CRABEquippedEntity*                    m_pcRABEquippedEntity;
00132       CWheeledEntity*                        m_pcWheeledEntity;
00133       CWiFiEquippedEntity*                   m_pcWiFiEquippedEntity;
00134    };
00135 
00136 }
00137 
00138 #endif
```

---

Generated on 10 Jul 2018 for ARGoS by 
 1.6.1
